# Investigating the Imperative Role of microRNAs Expression in Human Embryo Implantation: A Narrative Review Based on Recent Evidence

**DOI:** 10.3390/biomedicines12112618

**Published:** 2024-11-15

**Authors:** Anastasios Potiris, Sofoklis Stavros, Ioanna Zouganeli, Nikolaos Machairiotis, Eirini Drakaki, Athanasios Zikopoulos, Ismini Anagnostaki, Athanasios Zachariou, Angeliki Gerede, Ekaterini Domali, Peter Drakakis

**Affiliations:** 1 Third Department of Obstetrics and Gynecology, University General Hospital “ATTIKON”, Medical School, National and Kapodistrian University of Athens, 124 62 Athens, Greece; sfstavrou@med.uoa.gr (S.S.); joannazouga97@gmail.com (I.Z.); nikolaosmachairiotis@gmail.com (N.M.); thanzik92@gmail.com (A.Z.); isanagnostaki3@gmail.com (I.A.); pdrakakis@med.uoa.gr (P.D.); 2 First Department of Obstetrics and Gynecology, Alexandra Hospital, Medical School, National and Kapodistrian University of Athens, 115 28 Athens, Greece; eirinidrak@med.uoa.gr (E.D.); kdomali@yahoo.fr (E.D.); 3 Department of Urology, School of Medicine, Ioannina University, 451 10 Ioannina, Greece; zahariou@otenet.gr; 4 Department of Obstetrics and Gynecology, Democritus University of Thrace, 691 00 Alexandroupolis, Greece; agerede@otenet.gr

**Keywords:** microRNAs (miRNAs), embryo, implantation, endometrial receptivity, apposition, adhesion, invasion

## Abstract

Background/Objectives: Embryo implantation is a highly complex process that requires the precise regulation of numerous molecules to be orchestrated successfully. Micro RNAs (miRNAs) are small non-coding RNAs that regulate gene expression and play a crucial role in the regulation of embryo implantation. This article aims to summarize the key findings of the literature regarding the role of miRNAs in human embryo implantation, emphasizing their involvement in critical stages such as decidualization, endometrial receptivity and trophoblast adhesion. Methods: This review includes primary research articles from the past decade. The studies utilize a range of experimental methodologies, including gene expression analysis and in vitro studies. Results: MicroRNAs, like miR-320a, miR-149, and miR30d secreted by preimplantation embryos and blastocysts significantly influence endometrial receptivity by promoting essential cellular processes, such as cell migration and trophoblast cell attachment, while others—miR17-5p, miR-193-3p, miR-372, and miR-542-3p—secreted from the endometrium regulate the decidualization phase. During the apposition and adhesion phases, miRNAs play a complex role by promoting, for example, miR-23b-3p, and inhibiting—as do miR-29c and miR-519d-3p—important biological pathways of these stages. During invasion, miR-26a-5p and miR-125-5p modulate important genes. Conclusions: This review underscores the critical impact of miRNAs in the regulation of embryo implantation and early pregnancy. The ability of miRNAs to modulate gene expression at various stages of reproduction presents promising therapeutic avenues for improving assisted reproductive technologies outcomes and addressing infertility. Further research into miRNA-based diagnostic tools and therapeutic strategies is essential to enhance our understanding of their role in reproductive health and to exploit their potential for clinical applications.

## 1. Introduction

MicroRNAs (miRNAs) are small non-coding RNAs, typically about 22 nucleotides long, that regulate gene expression by binding to the 3′ untranslated regions of target messenger RNAs (mRNAs), leading to mRNA degradation or inhibition of translation [1]. Their sequences could be found in introns, inside host genes, or independently in the genome. A single miRNA could regulate more than 200 mRNAs [2]. They play critical roles in various biological processes, including embryo implantation, where they modulate the cross-talk between the embryo and the maternal endometrium [3]. The successful establishment of pregnancy hinges on the precise temporal and spatial regulation of endometrial receptivity, decidualization, and trophoblast invasion—all of which are tightly regulated by miRNAs [4]. These processes collectively ensure that the endometrium provides a supportive environment for the implanting embryo and that the trophoblast cells of the embryo can effectively invade and establish connections with the maternal blood supply, forming the basis of the placenta [5]. Dysregulation of miRNA expression during any of these stages can lead to reproductive complications such as infertility, recurrent pregnancy loss, and implantation failure, underscoring the significance of miRNAs in reproductive health [6,7,8,9,10].

Decidualization is a key event during pregnancy that involves the transformation of endometrial stromal cells into specialized decidual cells. This tissue change is driven by progesterone and begins during the luteal phase of the menstrual cycle, continuing after embryo implantation [11]. Decidual cells provide structural and nutritional support to the embryo and play a role in immune modulation to prevent maternal rejection of the embryo [12]. MiRNAs influence this process by regulating genes that are critical for the differentiation of stromal cells into decidual cells [13,14,15,16]. Dysregulation in miRNAs’ expression during this phase has been implicated in reproductive disorders and recurrent pregnancy loss [9,17,18].

Endometrial receptivity refers to the period during which the endometrium becomes favorable for blastocyst implantation, known as the window of implantation (WOI) [19]. This brief period typically occurs between days 20 and 24 of a regular menstrual cycle, and through this period, the endometrial lining undergoes changes that make it receptive [20]. Various molecules, including miRNAs, play essential roles in regulating these changes, influencing the expression of genes involved in cell adhesion, immune modulation, and cellular communication [17,21,22]. Specific miRNAs have been shown to regulate endometrial epithelial cell adhesion, establishing a connection between the embryo and the uterine lining [23,24]. Dysregulation of those miRNAs can impair endometrial receptivity, leading to implantation failure [22,25].

The process of embryo implantation is a complex series of steps that involves apposition, adhesion, and invasion. During the apposition phase, the blastocyst makes initial contact with the endometrial lining, usually at a site where the uterine lining has shed its outermost layer. This is followed by the adhesion phase, in which stronger connections are formed between the blastocyst and the endometrial cells through interactions mediated by adhesion molecules like integrins and cadherins [26,27]. MiRNAs play a crucial role in regulating the expression of these molecules, facilitating the firm attachment of the blastocyst to the uterine lining [23,28,29]. Finally, in the invasion phase, the trophoblast cells of the embryo penetrate the endometrial layer, allowing the establishment of a blood supply and the development of the placenta [30,31]. MiRNAs are also involved in regulating trophoblast invasion by targeting genes that control cellular migration, invasion, and proliferation [32,33].

Successful implantation of the embryo requires a well-coordinated balance of maternal and embryonic factors [34]. MiRNAs secreted by both the endometrium and the blastocyst play a significant role in modulating this balance by influencing gene expression in both tissues [35,36]. For example, miR-30d, secreted by the endometrium, has been shown to be taken up by the pre-implantation embryo, suggesting that endometrial miRNAs can modify embryonic gene expression to support implantation [30]. Conversely, miRNAs secreted by the blastocyst, such as miR-519d-3p, can negatively regulate endometrial epithelial cell adhesion, fine-tuning the interaction between the embryo and the maternal tissue [23].

**Figure 1:**
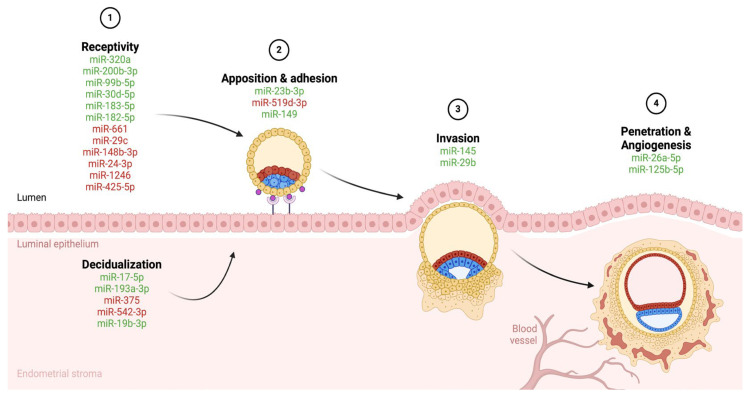
Schematic illustration of the expression and interaction of the studied miRNAs in the different phases of the blastocyst implantation process. Upregulated expression is marked with green and downregulated expression with red.

Ultimately, miRNAs are essential regulators of decidualization, endometrial receptivity, and embryo implantation. Their roles in modulating gene expression during these processes are crucial for reproductive success, and their dysregulation is linked to implantation failure and early pregnancy loss [37,38,39,40]. The aim of this review is to enlighten the specific functions of miRNAs in these contexts and provide new insights into the mechanisms of implantation offering potential therapeutic targets for improving fertility outcomes. Figure 1 illustrates the upregulated and downregulated miRNAs identified in this review intervening in different phases of embryo implantation process. Furthermore, Supplementary Table S1 includes the sequences of the studied miRNAs, Supplementary Table S2 presents the association of the included miRNAs with the stage of implantation and cell function and Supplementary Table S3 lists their source, target tissue and impact on implantation.

## 2. Literature Research

This review was conducted using peer-reviewed primary research articles published within the last decade (2014–2024) that focus on the role of microRNAs in human embryo implantation. A comprehensive literature search was performed using the PubMed/Medline and Scopus databases, employing keywords such as “microRNA”, “miRNA”, “human embryo implantation” and “embryo implantation” with the administration of Boolean operators (OR, AND) combined with those keywords either used as presented, separately and in combination. Studies included in the review were restricted to those utilizing human cells, tissues, or subjects to ensure clinical relevance. Only primary research articles with experimental data from human-derived samples, including endometrial tissues, trophoblasts, and human embryo cultures, were selected. Review articles, meta-analyses, animal models, and in silico studies were excluded. The search was further refined by including studies that directly investigated the molecular pathways involving microRNAs during embryo implantation. Additionally, the “snowball literature searching method” was applied to identify further relevant sources from the reference lists of selected articles.

Regarding quality assessment and risk of bias assessment of the included studies, a critical evaluation of each study’s sample (size of the sample), methodology (study design and compared groups), outcome presentation (clarity and relevance of reporting outcomes) and confounding factors (potential biases) was performed. This critical evaluation helped in the interpretation of each study’s outcomes and better presentation of our results and discussion on the effect of miRNAs on embryo implantation. A formal risk of bias and quality assessment was not performed due to the narrative nature of this review.

## 3. The Role of microRNAs in Endometrial Receptivity

Endometrial receptivity, often referred to as the ‘window of implantation’, is a brief period in the mid-luteal phase when the endometrium allows the blastocyst to be implanted [41]. Understanding the involvement of miRNAs in modulating this receptive state provides insights into the initial interactions between the embryo and the maternal endometrium. Table 1 summarizes the key findings of this review regarding the role of maternal miRNAs in endometrial receptivity and Table 2 the relevant fetal miRNAs.

To begin with, Berkhout et al. demonstrated that hsa-miR-320a, secreted by high-quality human preimplantation embryos, plays a crucial role in enhancing endometrial receptivity by promoting the migration of endometrial stromal cells. The research team utilized conditioned media collected from these embryos to treat endometrial stromal cells and observed an increase in cell migration. Synthetic miRNA mimics of hsa-miR-320a were also used to strengthen the results, which consistently replicated the enhanced migratory response [42]. Mechanistically, hsa-miR-320a was shown to target and downregulate PTEN, a well-studied tumor suppressor gene involved in inhibiting cell motility among other functions. The downregulation of PTEN leads to the activation of the PI3K/AKT signaling pathway, promoting cytoskeletal rearrangements and cellular motility [43]. Hence, embryos may actively secrete hsa-miR-320a to create a conducive microenvironment by enhancing the migratory capacity of endometrial stromal cells, thereby optimizing endometrial receptivity for implantation [42].

Similarly, the role of microRNAs secreted by human blastocysts in regulating endometrial epithelial cell adhesion was examined by Cuman et al. Human blastocysts were cultured, and their secreted miRNAs were identified using RNA sequencing. Human endometrial epithelial cells were then treated with these miRNAs, and adhesion assays were performed to measure changes in cell adhesive capacity. The study found that specific miRNAs, such as miR-320, significantly enhanced the adhesion of endometrial epithelial cells, while other miRNAs, like miR-661, secreted by non-implanted blastocysts, reduced the adhesion capacity of the trophoblast. This indicates that blastocysts actively secrete miRNAs to improve the likelihood of implantation by modulating the receptivity of the endometrium [28].

In contrast, Griffiths et al. explored the role of miR-29c in reducing endometrial epithelial cell adhesion, indicating a potential connection to infertility. Human endometrial epithelial cells were transfected with miR-29c mimics or inhibitors, and their adhesion capacity was evaluated using adhesion assays. The researchers also measured the expression of COL4A1, a gene involved in cell adhesion, using qPCR and Western blotting. They found that miR-29c overexpression downregulated COL4A1, leading to reduced adhesion of endometrial cells. This suggests that miR-29c might contribute to poor endometrial receptivity in infertile women, making it a potential biomarker for assessing implantation issues [44].

These findings were complemented by Ibáñez-Perez et al., who focused on identifying microRNA signatures in endometrial fluid that correlate with endometrial receptivity. Endometrial fluid samples were collected from women undergoing fertility treatments, and miRNA profiling was conducted using high-throughput sequencing. The researchers identified a set of miRNAs, including miR-30d, that were significantly associated with a receptive endometrium. These miRNAs were further validated through functional assays in vitro. The research team concluded two predictive models utilizing polymerase-based precipitation. The first model showed that hsa-miR-148-3p and hsa-miR-24-3p were both downregulated in the implantative endometrium. On the other hand, the second model showed that the upregulation of hsa-miR-200b-3p and hsa-miR-99b-5p was associated with a receptive endometrium. The results suggest that miRNA signatures from endometrial fluid could serve as non-invasive biomarkers to assess the receptivity of the endometrium, potentially improving implantation rates in assisted reproductive technologies (ART) [21].

Building upon this, researchers explored the interaction between the human endometrium and the preimplantation embryo, focusing on the role of hsa-miR-30d. Endometrial secretions containing hsa-miR-30d were collected from women, and human embryos were cultured with this miRNA. Transcriptomic analysis was then performed on the embryos to identify changes in gene expression. The study revealed that hsa-miR-30d is taken up by the blastocyst, where it modifies the embryo’s transcriptome. This suggests that miRNAs secreted by the endometrium play an active role in preparing the embryo for implantation by influencing its gene expression profile [33].

Regarding the role of adenomyosis in fertility, Juarez Barber, et al. aimed to explore the miRNA content of extracellular vesicles (EVs) secreted by endometrial organoids from adenomyosis patients and their role in embryo implantation. Endometrial organoids were cultured from endometrial biopsies of adenomyosis patients, and the secreted EVs were isolated and characterized. MiRNA sequencing was performed to analyze the miRNA content of the EVs. Additionally, in vitro experiments were conducted to assess the effect of these EVs on endometrial stromal cell function and embryo attachment. It was found that the EVs which were secreted contained miRNAs involved in implantation, including miR-1246 and miR-425-5p, which were shown to affect endometrial receptivity by modulating genes involved in cell adhesion and immune response. In vitro, the EVs from adenomyosis organoids impaired embryo attachment to endometrial cells, suggesting a potential mechanism by which adenomyosis may contribute to implantation failure [45].

Another study that aimed to find a relation between miRNA and endometrial receptivity was that of Akbar et al. They aimed to determine the role of miR-183-5p in regulating uterine receptivity and enhancing embryo implantation. Endometrial biopsies were collected from women during the implantation window, and the expression of miR-183-5p was assessed using qRT-PCR. In vitro experiments were performed on endometrial stromal cells to examine the effects of miR-183-5p overexpression and knockdown on cell proliferation, migration, and adhesion. Additionally, mouse models were used to evaluate the effect of miR-183-5p on embryo implantation. The study demonstrated that miR-183-5p enhances endometrial receptivity by promoting cell proliferation and adhesion. Overexpression of miR-183-5p increased integrin expression and improved embryo attachment in vitro, while knockdown of miR-183-5p impaired these processes. In vivo, mice treated with miR-183-5p showed improved implantation rates. So, it is suggested that miR-183-5p plays a critical role in regulating endometrial function and could be a potential target for improving implantation outcomes in assisted reproduction [46].

Yu et al. investigated the role of the miR-182-5p/NDRG1 axis in controlling endometrial receptivity via the NF-κB/ZEB1/E-cadherin pathway. Endometrial biopsies were obtained from women during the mid-luteal phase. qRT-PCR and Western blotting were used to measure miR-182-5p and its target gene NDRG1. In vitro experiments on endometrial epithelial cells were conducted to assess the impact of miR-182-5p on cell adhesion and gene expression in the NF-κB pathway. The study revealed that miR-182-5p downregulates NDRG1, leading to the activation of the NF-κB pathway and increased expression of ZEB1, which subsequently reduces E-cadherin levels. This downregulation of E-cadherin impairs cell–cell adhesion and endometrial receptivity. According to these findings, the miR-182-5p/NDRG1 axis could offer potential targets for improving endometrial receptivity in infertility treatments [47].

**Table 1:** Selected studies regarding the role of maternal miRNAs in endometrial receptivity.

Study	Study Type	Sample	Compared Groups	Outcome
Barton S, et al. (2023) [24]	Experimental in vitro study	Human endometrial epithelial cells	Cells treated with miR-23b-3p mimics vs. controls	miR-23b-3p regulates cell adhesion
Griffiths M, et al. (2019) [44]	Experimental in vitro study	Human endometrial epithelial cells from infertile women	miR-29c overexpression group vs. control	miR-29c overexpression reduced cell adhesion, affecting endometrial receptivity
Ibañez-Perez J, et al. (2022) [21]	Cross-sectional study	Endometrial fluid samples from women undergoing fertility treatments	Implantative vs. non-implantative endometrium	Identified miRNA signatures from endometrial fluid that could indicate implantative endometrium (hsa-miR-200b-3p and hsa-miR-99b-5p upregulated—hsa-miR-148-3p and hsa-miR-24-3p downregulated)
Vilella F, et al. (2015) [33]	Experimental in vitro study	Human endometrium and preimplantation embryos	Treated vs. untreated embryos	miR-30d, secreted by the endometrium, is taken up by embryos, potentially altering their transcriptome
Juárez-Barber E, et al. (2023) [45]	Experimental study	Adenomyosis endometrial organoids	Organoids with/without miRNAs in extracellular vesicles	Extracellular vesicles from adenomyosis endometrial organoids contain miRNAs involved in embryo implantation
Akbar R, et al. (2020) [46]	Experimental in vitro study	Uterine tissues and embryos	miR-183-5p overexpression vs. control	miR-183-5p enhances embryo implantation by regulating uterine receptivity
Yu SL, et al. (2022) [47]	Experimental study	Endometrial tissues	miR-182-5p overexpression vs. control	miR-182-5p/NDRG1 axis controls endometrial receptivity via the NF-κB/ZEB1/E-cadherin pathway

**Table 2:** Selected studies regarding the role of fetal miRNAs in endometrial receptivity.

Study	Study Type	Sample	Compared Groups	Outcome
Berkhout RP, et al. (2020) [42]	Experimental in vitro study	Human preimplantation embryos and endometrial stromal cells	High-quality embryos vs. low-quality embryos	High-quality embryos secrete miR-320a, stimulating endometrial stromal cell migration
Cuman C, et al. (2015) [28]	Experimental in vitro study	Human blastocysts and endometrial epithelial cells	Cells treated with microRNAs vs. controls	microRNAs secreted by blastocysts and especially miR-661 regulate endometrial epithelial cell adhesion

## 4. The Role of microRNAs in Endometrial Decidualization

While endometrial receptivity sets the stage for successful implantation, the process does not end there. Following the establishment of receptivity, the endometrium undergoes decidualization, a process in which endometrial glands transform into secretory ones, immune response modulates, new blood vessels are formed, and endometrial stromal cells become specialized epitheloid and miRNAs that help in the regulation [48].

For the part of miRNAs in decidualization to be analyzed, Soczewski et al. investigated the relationship between miRNAs and endoplasmic reticulum (ER) stress and the unfolded protein response (UPR) during the process of transformation of human endometrial stromal cells. Decidualization was induced in endometrial stromal cells using cyclic adenosine monophosphate (cAMP) and medroxyprogesterone acetate (MPA), simulating the hormonal conditions of early pregnancy. The team used miRNA sequencing to identify differentially expressed miRNAs during decidualization under normal and ER-stress conditions. Western blotting and qPCR were employed to analyze the expression of UPR-related proteins and mRNAs. The study found that specific miRNAs, including miR-17-5p and miR-193-3p, were significantly associated with the regulation of ER stress and the UPR during decidualization. These miRNAs were shown to regulate key UPR components, such as ATF6 and XBP1, which are crucial for maintaining cellular homeostasis during decidualization. Therefore, it is suggested that miRNAs play an important role in balancing ER stress and promoting proper decidualization, an essential step for pregnancy success [49].

In addition to regulating ER stress, miRNAs also target other pathways crucial for decidualization, as demonstrated by Yu et al., who focused on the role of hsa-miR-375 in suppressing NOX4 during this process. Furthering this, the impact of hsa-miR-375 in decidualization was examined on NADPH oxidase 4 (NOX4), a gene critical for decidualization of human endometrial stromal cells. Decidualization was induced in endometrial stromal cells using cAMP and MPA. The researchers employed miRNA mimics and inhibitors to manipulate the expression of hsa-miR-375 in these cells. Decidualization markers, such as prolactin and IGFBP-1, were assessed using enzyme-linked immunosorbent assays (ELISA), and qPCR and Western blotting were used to measure NOX4 expression levels, an enzyme that modulates reactive oxygen species (ROS) during cellular differentiation. The study found that overexpression of hsa-miR-375 inhibited the decidualization of endometrial stromal cells by targeting NOX4, leading to a significant reduction in ROS expression. This decrease in ROS impaired the activation of downstream signaling pathways, including the MAPK and PI3K/AKT pathways, which are crucial for the upregulation of decidualization-specific genes. Conversely, inhibition of hsa-miR-375 enhanced NOX4 expression, increased ROS levels, and promoted the decidualization process. Concluding, hsa-miR-375 acts as a negative regulator of decidualization by modulating the redox environment of endometrial stromal cells, thereby influencing their capacity to support early pregnancy. MiR-375 targets NOX4, resulting in modulating oxidative stress responses and influencing the decidualization process. That is why miR-375 is recognized as a key player in maintaining the delicate balance required for successful implantation and pregnancy [50].

Moreover, Qu et al. provided detailed insights into the regulatory role of miR-542-3p in decidualization by targeting the integrin-linked kinase (ILK) pathway, which plays a pivotal role in cell–matrix interactions and intracellular signaling. During decidualization, the transformation of endometrial stromal cells into decidual cells involves extensive cytoskeletal reorganization and changes in cell adhesion properties. The ILK pathway is critical in these processes as it integrates signals from the extracellular matrix through interactions with integrins, thereby modulating cellular responses such as proliferation, migration, and differentiation. It was demonstrated that miR-542-3p directly targets the 3′ untranslated region (UTR) of ILK mRNA, leading to its downregulation. This suppression of ILK disrupts downstream signaling cascades, including the activation of AKT and ERK pathways, which are essential for the upregulation of decidualization markers like prolactin and IGFBP-1. Consequently, overexpression of miR-542-3p resulted in reduced levels of these markers, indicating impaired decidualization. In contrast, inhibition of miR-542-3p led to increased ILK expression, enhanced AKT/ERK activation, and a corresponding upregulation of decidualization markers. This regulatory mechanism highlights the role of miR-542-3p as a potential negative regulator of decidualization, where its dysregulation could contribute to pathologies such as recurrent implantation failure and infertility. Understanding these pathways provides valuable insights into the complex regulatory networks governing endometrial transformation and suggests potential therapeutic targets for improving endometrial receptivity and implantation success. Decidualization was induced in endometrial stromal cells using a combination of cAMP and MPA. The researchers used miR-542-3p mimics and inhibitors to manipulate miRNA expression, and ILK pathway activity was assessed using Western blotting and qPCR. Decidualization markers, such as prolactin and IGFBP-1, were also measured to determine the extent of decidualization. The conclusion was that overexpression of miR-542-3p significantly suppressed the decidualization process by downregulating the expression of ILK and its downstream signaling components, including AKT and ERK. Conversely, inhibition of miR-542-3p enhanced ILK pathway activity and promoted decidualization, as evidenced by increased levels of decidualization markers. The research team concluded that miR-542-3p acts as a negative regulator of decidualization by targeting the ILK pathway, potentially contributing to conditions where decidualization is impaired, such as infertility or early pregnancy loss [14].

Together, these findings highlight the diverse mechanisms through which miRNAs influence decidualization, further underscored by Menkhorst et al., who identified a critical reduction in miR-19b-3p release during decidualization. The release of miR-19b-3p by endometrial stromal cells during decidualization and its role in decidual-trophoblast cross-talk were explored. Endometrial stromal cells were isolated from biopsies and induced to decidualize in vitro. qRT-PCR was used to measure the expression and release of miR-19b-3p. Coculture assays with trophoblast cells were conducted to assess the impact of miR-19b-3p on trophoblast invasion and adhesion. During decidualization it was found that the production of miR-19b-3p by endometrial stromal cells was significantly reduced. This reduction impaired decidual-trophoblast cross-talk, as evidenced by decreased trophoblast invasion in coculture assays. Thus, it is suggested that miR-19b-3p takes a leading role in mediating communication between the decidua and trophoblasts, which is critical for proper placental development and embryo implantation [13]. Table 3 presents the key findings of this review regarding the role of miRNAs in endometrial decidualization.

## 5. The Role of miRNAs in Apposition–Adhesion

Once the endometrium has been prepared through receptivity and decidualization, the next critical steps in implantation involve the apposition and adhesion of the blastocyst to the uterine lining and these phases require precise cellular interactions, mediated by a variety of molecular signals, including miRNAs [51].

The role of miR-23b-3p in regulating human endometrial epithelial cell adhesion was investigated by Barton et al. Human endometrial epithelial cells (HEECs) were isolated from endometrial biopsies and subjected to miRNA transfection to either overexpress or inhibit miR-23b-3p. Adhesion assays were conducted to assess the cells’ ability to adhere to the extracellular matrix (ECM). Additionally, quantitative PCR (qPCR) and Western blotting were performed to analyze the expression of target genes influenced by miR-23b-3p. The results demonstrated that overexpression of miR-23b-3p significantly enhanced HEEC adhesion to the ECM. The study also identified genes that miR-23b-3p targets, offering insights into how this miRNA regulates cell adhesion and potentially improves endometrial receptivity for embryo implantation [24].

Conversely, Wang et al. provided insights into the role of miR-519d-3p, a miRNA secreted by human blastocysts, in modulating the adhesion of endometrial epithelial cells during the critical phases of implantation. MiR-519d-3p was shown to directly target hypoxia-inducible factor 1-alpha (HIF1α), a transcription factor that plays a pivotal role in cellular responses to hypoxic conditions often present in the endometrium during the window of implantation. HIF1α is known to regulate the expression of various adhesion molecules, such as E-cadherin and integrins, which facilitate the attachment of the blastocyst to the uterine lining. The study revealed that miR-519d-3p downregulates HIF1α, leading to a decrease in E-cadherin expression. This reduction in E-cadherin disrupts cell–cell junctions and weakens the adhesive properties of endometrial epithelial cells, thereby fine-tuning the endometrial receptivity to ensure that only high-quality blastocysts are able to initiate successful implantation. This miRNA-mediated modulation of adhesion provides a mechanism for selective implantation, potentially preventing the attachment of embryos with lower viability. Human endometrial epithelial cells (HEECs) and blastocysts cultured in vitro were utilized. miRNA expression was manipulated using miR-519d-3p mimics and inhibitors, while HIF1α expression was measured through quantitative PCR (qPCR) and Western blotting. Adhesion assays were conducted to evaluate the ability of HEECs to adhere to the extracellular matrix (ECM) under varying conditions of miR-519d-3p expression. Additionally, luciferase reporter assays were used to confirm that HIF1α was a direct target of miR-519d-3p. It was concluded that miR-519d-3p, secreted by human blastocysts, significantly reduced the adhesion of endometrial epithelial cells to the ECM. This was shown to occur through the downregulation of HIF1α, an essential factor involved in cell adhesion and implantation processes. Overexpression of miR-519d-3p resulted in decreased HIF1α levels and reduced adhesion, whereas inhibition of miR-519d-3p had the opposite results. Following the logic order, miR-519d-3p plays a negative regulatory role in endometrial receptivity by targeting HIF1α, and its dysregulation could potentially contribute to implantation failures [23].

In addition to these findings, Soni et al. investigated the role of miR-149 in regulating the attachment of trophoblast cells to the endometrial lining. Human endometrial epithelial and stromal cells were treated with miR-149 mimics and inhibitors, and trophoblast attachment assays were performed. The researchers also measured the expression of PARP-2, a gene targeted by miR-149, through qPCR and Western blotting. As a result, miR-149 downregulated PARP-2, promoting the attachment of trophoblast cells, defining this miRNA’s key role in enhancing trophoblast adhesion, which is essential for successful implantation [52]. Table 4 summarizes the outcomes of the included studies regarding apposition and adhesion in this review.

## 6. The Role of microRNAs in Blastocyst Invasion and Placental Formation

Successful adhesion of the blastocyst to the endometrium marks the beginning of the invasive phase, where trophoblast cells penetrate the maternal tissues to establish a connection with the maternal blood supply. The trophoblast layer, which forms the outer layer of the blastocyst, is responsible for the implantation into the uterine wall and the formation of the placenta, making its proper function vital for a healthy pregnancy. This invasion is crucial for the formation of the placenta, which supports the developing fetus throughout pregnancy [53]. The role of miRNAs in this process is multifaceted, influencing trophoblast invasion, migration, and differentiation. Table 5 highlights the main outcomes of the included studies in this section.

Focusing on blastocyst invasion and placental formation, Eivazi et al. studied the role of miRNAs secreted by human blastocysts as potential regulators of gene expression during the implantation process. Human blastocysts were cultured, and their conditioned media was collected. The miRNAs present in the media were identified using high-throughput sequencing. Endometrial epithelial and stromal cells were then treated with these miRNAs, and the expression of target genes involved in implantation was analyzed using quantitative PCR (qPCR) and Western blotting. Functional assays, such as cell adhesion and invasion assays, were performed to assess the impact of miRNAs on endometrial cell behavior. Several miRNAs, including miR-145 and miR-29b, which were secreted by blastocysts, were identified as playing a regulatory role in gene expression related to endometrial receptivity and implantation. These miRNAs were shown to influence key processes, such as cell adhesion and invasion, by modulating the expression of genes like ITGB3 and MMP9. This suggests that miRNAs secreted by blastocysts may act as signaling molecules to enhance endometrial receptivity, facilitating successful implantation [54].

Similarly, Szuszkiewicz et al. investigated the effects of miR-26a-5p and miR-125b-5p on trophoblast cells, focusing on their regulation of genes critical for trophoblast function during early pregnancy. Human trophoblast cell lines were transfected with miR-26a-5p and miR-125b-5p mimics and inhibitors. Gene expression changes were analyzed using qPCR, and Western blotting was performed to measure the expression of proteins involved in trophoblast invasion, adhesion, and differentiation. Functional assays, such as invasion, migration, and proliferation assays, were conducted to assess how these miRNAs impact trophoblast behavior. It was revealed that miR-26a-5p and miR-125b-5p regulate several genes involved in trophoblast function, including VEGFA, MMP2, and GATA2. Specifically, miR-26a-5p was found to downregulate the expression of VEGFA (vascular endothelial growth factor A), a critical factor that promotes angiogenesis and supports trophoblast invasion by enhancing angiogenesis. Similarly, miR-125b-5p was demonstrated to regulate the expression of MMP2 (matrix metalloproteinase 2), an enzyme involved in the degradation of extracellular matrix components, facilitating the penetration of trophoblast cells into the maternal endometrium. Furthermore, the study highlighted the role of GATA2, a transcription factor essential for trophoblast differentiation, as another target of miR-125b-5p. By modulating GATA2 expression, miR-125b-5p influences the differentiation of trophoblast cells into specialized subtypes required for proper placental development. Overexpression of these miRNAs reduced trophoblast invasion and migration, while their inhibition enhanced these processes. As a result, miR-26a-5p and miR-125b-5p seem to play important roles in controlling trophoblast function during early pregnancy, and dysregulation of these miRNAs may contribute to pregnancy complications such as preeclampsia and recurrent miscarriage [32].

Collectively, these studies illustrate the multifaceted role of miRNAs across various stages of implantation. However, their potential extends beyond molecular regulation, as they also present promising avenues for clinical intervention, especially in conditions like implantation failure and pregnancy loss. While the molecular mechanisms underlying implantation have become clearer, the clinical implications of miRNAs in diagnosing and treating reproductive disorders are equally promising, as evidenced by recent research on their potential use as biomarkers and therapeutic targets [55,56].

## 7. Discussion

Recent studies have highlighted the significant role of microRNAs in regulating the critical process of endometrial receptivity. High-quality human preimplantation embryos secrete hsa-miR-320a, which in turn stimulates the migration of endometrial stromal cells [42]. The role of miR-23b-3p has emerged in modulating the adhesive properties of endometrial epithelial cells, ensuring that the endometrial surface is optimally prepared for embryo attachment [24]. In addition to miRNAs produced by the endometrium itself, blastocyst-derived miRNAs regulate the adhesion of endometrial epithelial cells, thus directly affecting the endometrial environment’s receptivity to implantation [28].

On the contrary, the expression of miR-29c in the endometrium has been linked to reduced epithelial cell adhesion in cases of infertility, leading to the conclusion that this could be used as a biomarker for infertile women [44]. Moreover, specific miRNA-based signatures offer potential biomarkers for assessing endometrial receptivity and may guide personalized treatments in assisted reproductive technologies [21].

Specific miRNAs could potentially serve as therapeutic targets in assisted reproductive medicine, aiming to improve the local uterine environment and increase the likelihood of implantation and pregnancy. As an example, miR-183-5p improves embryo attachment in vitro, so it could be utilized to enhance implantation outcomes [46]. On the other hand, miR-182-5p was shown to downregulate the process of implantation and could be used as a target for improving receptivity in fertility treatments [47].

It is indicated that endometrial miRNAs can influence embryonic development even before implantation, as it was indicated that hsa-miR-30d, secreted by the endometrium, is taken up by the pre-implantation embryo, potentially modifying its transcriptome. These data support the concept that miRNAs play a multifaceted role in ensuring successful implantation [33].

Endometrial decidualization is a crucial process in the establishment and maintenance of pregnancy. Recent research has shown that during decidualization, the proper function of miRNAs and unfolded protein response—a cellular response to endoplasmic reticulum stress maintaining homeostasis—in endometrial stromal cells is necessary for supporting cell survival and differentiation [49]. Also, it was shown that overexpression of hsa-miR-375 in endometrial stromal cells resulted in the suppression of NOX4 expression, leading to a significant reduction in ROS production and decidualization markers. These findings suggest that hsa-miR-375 negatively regulates decidualization by directly suppressing NOX4, making it a potential target for improving endometrial receptivity in conditions such as recurrent implantation failure [50]. Additionally, it was discovered that by modulating ILK pathways, miR-542-3p affects the ability of endometrial stromal cells to undergo proper decidualization, thereby influencing implantation outcomes [14]. The communication between trophoblast and deciduae could be disrupted as a well by the lack of miR-19b-3p, resulting to inadequate blastocyst invasion [13]. Collectively, these studies underscore the critical role of miRNAs in regulating endometrial decidualization and, consequently, implantation. Disruption of this balance due to aberrant miRNA expression can impair the decidualization process and potentially affect implantation success [49]. Understanding these regulatory mechanisms highlights potential therapeutic targets for addressing implantation disorders.

The process of implantation involves a series of coordinated steps that enable the embryo to attach to the uterine lining and establish a pregnancy. As we have seen before, miR-23b-3p regulates human endometrial epithelial cell adhesion, by modulating the expression of specific molecules mediating the initial attachment of the embryo [24]. Certain miRNAs, including miR-661 and others, are secreted by the blastocyst and act on the endometrial epithelial cells to modulate their adhesion properties. This interaction is essential for the proper apposition of the embryo to the uterine lining, a precursor to firm adhesion and invasion [28]. Furthermore, by downregulating HIF1α, miR-519d-3p—which is released by human blastocysts—reduces the expression of adhesion molecules, suggesting that miR-519d-3p may play a role in fine-tuning the receptivity of the endometrium to ensure that implantation occurs only under optimal conditions [23]. Moreover, miRNA-149 has been implicated in regulating trophoblast attachment, a key step in embryo implantation. MiRNA-149 targets PARP-2 in both endometrial epithelial and stromal cells, thereby modulating the trophoblast’s ability to attach and invade the endometrial tissue [52]. Overall, these studies underscore the importance of miRNAs in the regulation of endometrial epithelial cell behavior during the early stages of implantation. By modulating the expression of key adhesion molecules, miRNAs influence both the apposition and adhesion phases, which are crucial for successful implantation.

In the process of implantation, it is demonstrated that miRNAs, as miR-145 and miR-29b secreted by human blastocysts have the potential to regulate gene expression in the endometrial tissue, influence the early stages of implantation. These miRNAs likely contribute to preparing the endometrium for the embryo’s attachment and subsequent invasion [54].

Beyond the early implantation phase, miRNAs also play a role in trophoblast function and placental formation. The roles of miR-26a-5p and miR-125b-5p in the regulation of trophoblast function were investigated and they were shown to target several key genes involved in trophoblast cell invasion, migration, and differentiation. The suppression of VEGFA by miR-26a-5p leads to reduced trophoblast proliferation and invasion, potentially contributing to complications such as preeclampsia, where inadequate placental development is a hallmark. Overexpression of miR-125b-5p led to a decrease in MMP2 levels, impairing the invasive capacity of trophoblasts and potentially contributing to disorders like fetal growth restriction (FGR) and recurrent miscarriage. These miRNAs are crucial for the proper differentiation and functioning of trophoblast cells, which are essential for the development of the placenta [32].

In summary, miRNAs secreted by blastocysts are critical regulators of both the implantation process and placental formation. They facilitate endometrial adhesion, trophoblast function, and overall placental development, highlighting their potential as targets for therapeutic interventions in cases of implantation failure and placental dysfunction.

## 8. Limitations and Challenges in miRNA Research

Despite significant advancements in current research studies of miRNAs in human embryo implantation, several critical limitations persist, hindering a comprehensive elucidation of their functions and translational potential in clinical practice.

The first, and probably the most important, difficulty in miRNA research is the heterogeneity in experimental designs and methodologies across different studies. The great differences in sample types, the timing of sample collection relative to menstrual cycle, and miRNA detection techniques result in inconsistent data and reduced reproducibility [15,16,17]. Moreover, uncommonly applied criteria for terms such as “successful” implantation or “receptive” endometrium led to discrepancies in study outcomes and impediment of generalization [13,22]. Adding to that, there are not standardized criteria for miRNA biomarker validation, neither is there a consensus on which miRNA should be utilized in clinical practice [21,33]. As an addition, studies often suffer from small sample sizes and different patient populations, including varying age ranges, underlying reproductive disorders, and treatment histories, for example therapies of ART [17,22]. This variability can introduce confounding factors that obscure the true effects of miRNAs on implantation [15,17,22,47]. Additionally, scientists mostly rely on in vitro models or animal studies, which, although informative, are inadequate to capture the complexity of human implantation and pose significant barriers due to differences in miRNA expression and functions in other species and the limitations of cell-line cultures [23,29]. Conducting research on human implantation poses ethical and practical challenges, particularly in obtaining suitable samples from the peri-implantation period without compromising the pregnancy. As a result, studies often rely on alternate samples, such as mid-luteal phase endometrial biopsies, which may not accurately reflect the in vivo conditions during implantation [13,50]. Moreover, the ability to manipulate miRNA levels in human subjects poses ethical considerations, obstructing the direct testing of hypotheses generated from in vitro or animal models [23,32]. Furthermore, there is a lack of longitudinal studies tracking miRNA expression and activity throughout the different phases of implantation, and therefore more such studies are essential for understanding the dynamic changes in miRNA-mediated regulation and their impact on implantation success or failure [49,54].

The expression and function of miRNAs are highly temporal and spatial dynamic, but many studies do not account for these dynamics, leading to potentially misleading conclusions [16,42,46,49]. Additionally, many studies primarily focus on miRNA expression profiles without performing in-depth functional analyses to confirm the biological significance of these findings, limiting our understanding of molecular mechanisms of implantation [14,50,52]. Although miRNAs function within complex regulatory networks, often targeting multiple genes and pathways, most studies tend to use a reductionist approach focusing on single miRNA–target interactions and oversimplifying the multifaceted roles of miRNAs in implantation [32,46]. For a more holistic understanding, future research should incorporate systems biology approaches to map the intricate networks of miRNA interactions and their cross-talk with other regulatory molecules, such as transcription factors, cytokines, and hormones [31,45]. Moreover, the dynamic nature of miRNA expression, influenced by hormonal fluctuations and environmental factors, poses additional challenges for their reliable use in diagnostics [42,44]. Despite promising preclinical findings, translating miRNA research into therapeutic applications faces significant hurdles. The multifunctionality and pleiotropy of miRNAs mean that modulating a single miRNA could have unintended consequences on multiple biological pathways [47,52]. Additionally, effective delivery systems that can specifically target miRNAs to the endometrium or trophoblast without off-target effects are still under development. Regulatory challenges and safety concerns further complicate the clinical translation of miRNA-based therapies [31,44]. Single-omics approaches, focusing solely on miRNA expression, provide a limited view of the complex biological processes involved in implantation, thus making the use of the other omics layers—such as transcriptomics, proteomics, and epigenomics—that are necessary in order to offer a more comprehensive understanding of the regulatory networks at play [21,24]. However, such integrative studies are currently scarce due to technical and analytical challenges, including data harmonization and the need for advanced computational tools [29,45].

## 9. Future Directions

Addressing these limitations through standardized protocols, robust experimental designs, and innovative methodologies will be essential for advancing our understanding of miRNA roles in implantation and their potential as therapeutic targets. Future research should focus on overcoming these challenges to unlock the full potential of miRNAs in improving reproductive health outcomes.

A major challenge lies in the complex regulatory nature of miRNAs. MiRNAs can target multiple genes, and each gene can be regulated by numerous miRNAs, leading to intricate molecular networks [14], making it challenging to predict the outcomes of manipulating specific miRNAs. Therefore, comprehensive studies to map miRNA–target networks in the context of implantation are necessary. Targeting miRNAs for therapeutic purposes holds promise but presents considerable challenges. Administration of miRNA mimics or inhibitors either with intrauterine catheterization or with local injection into the endometrial tissue or the peritoneal cavity has been tested in animal models for the treatment of endometriosis [57,58]. Extrapolating these results to humans and assessing these effects in the more complex field of assisted reproductive techniques needs further research. Furthermore, manipulating a single miRNA may lead to unintended effects on multiple biological pathways [23]. This redundancy and pleiotropy complicate the development of miRNA-based therapies, requiring highly targeted delivery systems to avoid off-target effects. Variations in miRNA expression across individuals and reproductive cycles present another hurdle. External factors such as hormonal fluctuations, environmental exposures, and stress can influence miRNA levels. This variability poses a significant challenge for developing miRNA-based diagnostic methods for clinical use, requiring personalized approaches to account for these fluctuations [21]. Genetic polymorphisms in miRNAs or their target genes can significantly impact their regulatory roles, and understanding of how such polymorphisms affect miRNA function is crucial for designing personalized treatments aimed at restoring normal implantation processes in individuals with these genetic variations [15,59,60].

Another field that could be used in ART therapy is cellular stress response. Further research is needed to elucidate the mechanisms by which stress-related miRNAs impact embryo implantation, with the aim of identifying therapeutic targets that could mitigate stress-induced implantation failure [49]. Extracellular vesicles (EVs) have been shown to carry miRNAs critical for embryo–endometrium communication. However, the precise mechanisms governing miRNA packaging into EVs, their transport, and their functional roles remain poorly understood. Developing clinical tools to harness EVs for miRNA delivery faces challenges related to the stability, isolation, and targeted delivery of these vesicles [54]. Finally, the development of miRNA-based diagnostic and therapeutic tools must navigate ethical and regulatory challenges. Concerns over the long-term effects of genetic manipulation, particularly in the context of reproductive health, require rigorous preclinical and clinical testing to ensure safety and efficacy. Moreover, the regulatory framework for implementing miRNA-based therapies in fertility clinics remains underdeveloped.

In conclusion, miRNAs present exciting opportunities for advancing reproductive medicine by offering new biomarkers and therapeutic targets. However, future research must address the complexity of miRNA regulation, variability in expression, genetic polymorphisms, and their roles in stress responses and RIF. By overcoming these challenges, miRNA-based strategies have the potential to significantly improve implantation outcomes and infertility treatments.

## 10. Conclusions

This review underscores the intricate ways in which miRNAs contribute to both promoting and hindering successful implantation, reflecting their complex regulatory functions in reproductive health. MicroRNAs secreted by preimplantation embryos and blastocysts significantly influence endometrial receptivity. Studies have shown that miRNAs such as hsa-miR-320a, miR-149, and miR-30d enhance endometrial receptivity by promoting essential cellular processes, such as cell migration and trophoblast cell attachment. On the other hand, some miRNAs, such as miR-29c and miR-519d-3p, act as negative regulators, by inhibiting adhesion. Research has demonstrated that miRNAs like miR-17-5p and miR-193a-3p are involved in managing endoplasmic reticulum stress during decidualization, leading to the maintenance of cellular homeostasis, which is vital for successful implantation. Furthermore, miR-375 negatively regulates decidualization by suppressing NOX4, a critical for this process enzyme, while miR-542-3p inhibits decidualization by targeting the integrin-linked kinase (ILK) pathway. During the apposition and adhesion phases, miRNAs play a complex role by promoting and inhibiting important biological pathways of the stages. miR-23b-3p, for example, enhances the adhesion of endometrial epithelial cells to the extracellular matrix. Conversely, miR-519d-3p downregulates key adhesion factors, indicating its inhibiting role in regulating endometrial receptivity. In the later stages of implantation, miRNAs also regulate trophoblast invasion and placental development. Studies have highlighted the roles of miR-26a-5p and miR-125b-5p in controlling trophoblast cell function by modulating genes involved in trophoblast invasion, migration, and differentiation. Overall, the evidence from these studies emphasizes the significant impact of miRNAs on various stages of reproduction. Their ability to regulate key processes involved in implantation and placental development presents promising avenues for improving clinical outcomes in assisted reproductive technologies and addressing reproductive disorders. Further research is essential to deepen our understanding of miRNA mechanisms and explore their potential as diagnostic tools and therapeutic targets in reproductive medicine.

## Figures and Tables

**Table 3:** Selected studies regarding the role of miRNAs in endometrial decidualization.

Study	Study Type	Sample	Compared Groups	Outcome
Soczewski E, et al. (2023) [49]	Experimental study	Decidualized endometrial stromal cells	Cells with induced endoplasmic reticulum stress vs. controls	Investigated the role of miRNAs in endoplasmic reticulum stress and unfolded protein response during decidualization
Yu SL, et al. (2022) [50]	Experimental study	Endometrial stromal cells	Cells overexpressing hsa-miR-375 vs. controls	hsa-miR-375 impairs decidualization by targeting NOX4
Qu X, et al. (2021) [14]	Experimental study	Endometrial stromal cells	miR-542-3p-treated cells vs. control	miR-542-3p suppresses decidualization by targeting the ILK pathway
Menkhorst E, et al. (2023) [13]	Experimental study	Endometrial stromal cells	Decidualized cells vs. non-decidualized controls	reduced miR-19b-3p release during decidualization, indicating a role in decidual-trophoblast cross-talk

**Table 4:** Selected studies regarding the role of miRNAs in apposition–adhesion.

Study	Study Type	Sample	Compared Groups	Outcome
Barton S, et al. (2023) [24]	Experimental in vitro study	Human endometrial epithelial cells	Cells treated with miR-23b-3p mimics vs. controls	miR-23b-3p regulates cell adhesion, implying a role in implantation
Cuman C, et al. (2015) [28]	Experimental in vitro study	Human blastocysts and endometrial epithelial cells	Cells treated with microRNAs vs. controls	microRNAs secreted by blastocysts regulate endometrial epithelial cell adhesion
Wang X, et al. (2022) [23]	Experimental study	Human blastocysts and endometrial epithelial cells	Cells exposed to miR-519d-3p vs. controls	miR-519d-3p, released by blastocysts, reduces endometrial cell adhesion by targeting HIF1α
Soni UK, et al. (2021) [52]	Experimental in vitro study	Endometrial epithelial and stromal cells	miRNA-149-treated cells vs. control cells	miR-149 targets PARP-2 to regulate trophoblast attachment

**Table 5:** Selected studies regarding the role of miRNAs in blastocyst invasion and placental formation.

Study	Study Type	Sample	Compared Groups	Outcome
Cuman C, et al. (2015) [28]	Experimental in vitro study	Human blastocysts and endometrial epithelial cells	90 women with recurrent implantation failure (RIF) and 40 fertile women	microRNAs secreted by blastocysts regulate endometrial epithelial cell adhesion
Eivazi S, et al. (2023) [54]	Experimental study	Human blastocysts	Blastocysts with secreted miRNAs vs. controls	miRNAs secreted by blastocysts could act as regulators during implantation
Szuszkiewicz J, et al. (2022) [32]	Experimental study	Trophoblast cells	Cells treated with miR-26a-5p and miR-125b-5p vs. controls	miR-26a-5p and miR-125b-5p affect genes and cell functions critical during early pregnancy

## Supplementary Materials

The following supporting information can be downloaded at: https://www.mdpi.com/article/10.3390/biomedicines12112618/s1, Table S1: miRNAs referred in the review and their sequences, Table S2: micro RNAs included in the review and association with the stage of implantation and cell function, Table S3: List of referred miRNAs, their source, their target tissue and impact on implantation.
