# Supplementary material for: Investigating the Imperative Role of microRNAs Expression in Human Embryo Implantation: A Narrative Review Based on Recent Evidence
Source: Biomedicines. 2024 Nov 15;12(11):2618. doi: 10.3390/biomedicines12112618 (PMC11592390; doi:10.3390/biomedicines12112618)
Supplement: Supplementary file 1 [file biomedicines-12-02618-s001.zip › biomedicines-3296343-supplementary.pdf]

Table S1. miRNAs referred in the review and their sequences.

miIRNA	Sequence	Reference
hsa-miR-320a-3p	42 - AAAAGCUGGGUUGAGAGGGCGA - 63	42
hsa-miR-320a-5p	10 - GCCUUCUCUUCCCGGUUCUUCC - 31	42
hsa-miR-661	UGCCUGGGUCUCUGGCCUGCGCGU	28
hsa-miR-29c-5p	16 - UGACCGAUUUCUCCUGGUGUUC - 37	44
hsa-miR-29c-3p	54 - UAGCACCAUUUGAAAUCGGUUA - 75	44
hsa-miR-148b-3p	UCAGUGCAUCACAGAACUUUGU	21
hsa-miR-24-3p	UGGCUCAGUUCAGCAGGAACAG	21
hsa-miR-200b-3p	UAAUACUGCCUGGUAAUGAUGA	21
hsa-miR-99b-5p	CACCCGUAGAACCGACCUUGCG	21
hsa-miR-30d-5p	UGUAAACAUCCCCGACUGGAAG	33
hsa-miR-1246	AAUGGAUUUUUGGAGCAGG	45
hsa-miR-425-5p	AAUGACACGAUCACUCCCGUUGA	45
hsa-miR-183-5p	UAUGGCACUGGUAGAAUUCACU	46
hsa-miR-182-5p	UUUGGCAAUGGUAGAACUCACACU	47
hsa-miR-17-5p	CAAAGUGCUUACAGUGCAGGUAG	50
hsa-miR-193a-3p	AACUGGCCUACAAAGUCCCAGU	50
hsa-miR-375-3p	40 - UUUGUUCGUUCGGCUCGCGUGA - 61	51
hsa-miR-375-5p	5 - GCGACGAGCCCCUCGCACAAACC - 27	51
hsa-miR-542-3p	UGUGACAGAUUGAUAACUGAAA	14
hsa-miR-19b-3p	UGUGCAAAUCCAUGCAAAACUGA	13
hsa-miR-23b-3p	AUCACAUUGCCAGGGAUUACCAC	24
hsa-miR-519d-3p	CAAAGUGCCUCCCUUUAGAGUG	23
hsa-miR-149-5p	15 - UCUGGCUCCGUGUCUUCACUCCC - 37	48
hsa-miR-149-3p	56 - AGGGAGGGACGGGGGCUGUGC - 76	48
hsa-miR-145-3p	16 - GUCCAGUUUUCCCAGGAAUCCCU - 38	54
hsa-miR-145-5p	54 - GGAUUCCUGGAAAUACUGUUCU - 75	54
hsa-miR-29b-3p	UAGCACCAUUUGAAAUCAGUGUU	54
hsa-miR-29b-1-5p	GCUGGUUUCAUAUGGUGGUUUAGA	54
hsa-miR-29b-2-5p	CUGGUUUCACAUGGUGGCUUAG	54
hsa-miR-26a-5p	UUCAAGUAAUCCAGGAUAGGCU	32
hsa-miR-125b-5p	UCCCUGAGACCCUAACUUGUGA	32

Table S2. micro RNAs included in the review and association with the stage of implantation and cell function.

MIRNA	STAGE	CELL FUNCTION	REFERENCE
hsa-miR-320a	Receptivity/ Adhesion	Cell migration	42
hsa-miR-661	Receptivity	Cell adhesion	28
hsa-miR-29c	Receptivity	Cell adhesion	44
hsa-miR-148b-3p	Receptivity		21
hsa-miR-24-3p	Receptivity		21
hsa-miR-200b-3p	Receptivity		21
hsa-miR-99b-5p	Receptivity		21
hsa-miR-30d-5p	Receptivity		33

hsa-miR-1246	Receptivity	Cell adhesion, immune response	45
hsa-miR-425-5p	Receptivity	Cell adhesion, immune response	45
hsa-miR-183-5p	Receptivity	Cell adhesion, proliferation	46
hsa-miR-182-5p	Receptivity	Cell adhesion	47
hsa-miR-17-5p	Decidualization	Regulation of endoplasmic reticulum stress and unfolded protein response, cellular homeostasis	50
hsa-miR-193a-3p	Decidualization	Regulation of endoplasmic reticulum stress and unfolded protein response, cellular homeostasis	50
hsa-miR-375	Decidualization	Modulates the redox environment of endometrial cells	51
hsa-miR-542-3p	Decidualization	Disturbs cell-matrix interactions and intracellular signaling	14
hsa-miR-19b-3p	Decidualization	Mediates decidual-trophoblast crosstalk	13
hsa-miR-23b-3p	Adhesion / receptivity	Cell adhesion	24
hsa-miR-519d-3p	Adhesion / receptivity	Cell adhesion	23
hsa-miR-149	Adhesion	Cell adhesion	48
hsa-miR-145	Invasion	Cell invasion/ adhesion	54
hsa-miR-29b	Invasion	Cell invasion/ adhesion	54
hsa-miR-26a-5p	Invasion	Angiogenesis	32
hsa-miR-125b-5p	Invasion	Penetration, differentiation	32

Table S3. List of referred miRNAs, their source, their target tissue and impact on implantation

MIRNA	SOURCE	TARGET TISSUE	IMPACT ON IMPLANTATION	REFERENCE
hsa-miR-320a	High-quality blastocyst	Endometrial stromal cells	Enhances endometrial receptivity, supports embryo adhesion	42
hsa-miR-661	Non – implanting blastocyst	Endometrial epithelial cells	Reduces receptivity	28
hsa-miR-29c	Endometrial epithelial cells	Endometrial epithelial cells	Reduces receptivity	44
hsa-miR-148b-3p	Endometrial cells	Endometrial cells	Reduces receptivity	21
hsa-miR-24-3p	Endometrial cells	Endometrial cells	Reduces receptivity	21
hsa-miR-200b-3p	Endometrial cells	Endometrial cells	Enhances receptivity	21
hsa-miR-99b-5p	Endometrial cells	Endometrial cells	Enhances receptivity	21
hsa-miR-30d-5p	Endometrial cells	Endometrial cells	Enhances endometrial receptivity, supports embryo adhesion	33
hsa-miR-1246	Endometrial cells	Endometrial cells	Reduces receptivity	45
hsa-miR-425-5p	Endometrial cells	Endometrial cells	Reduces receptivity	45
hsa-miR-183-5p	Endometrial cells	Endometrial cells	Enhances receptivity	46
hsa-miR-182-5p	Endometrial cells	Endometrial cells	Enhances receptivity	47
hsa-miR-17-5p	Endometrial cells	Endometrial cells	Enhances decidualization	50

hsa-miR-193a-3p	Endometrial cells	Endometrial cells	Enhances decidualization	50
hsa-miR-375	Endometrial cells	Endometrial cells	Reduces decidualization	51
hsa-miR-542-3p	Endometrial cells	Endometrial cells	Reduces decidualization	14
hsa-miR-19b-3p	Endometrial stromal cells	Endometrial cells and blastocyst	Mediated fetal-maternal crosstalk, promotes decidualization	13
hsa-miR-23b-3p	Endometrial epithelial cells	Endometrial cells	Increases adhesion and receptivity	24
hsa-miR-519d-3p	Blastocyst	Endometrium	Reduces adhesion and endometrial receptivity	23
hsa-miR-149	Blastocyst	Endometrium	Increases blastocyst attachment	48
hsa-miR-145	Blastocyst	Endometrium	Enhances blastocyst invasion and endometrial receptivity	54
hsa-miR-29b	Blastocyst	Endometrium	Enhances blastocyst invasion and endometrial receptivity	54
hsa-miR-26a-5p	Blastocyst	Endometrium	Enhances blastocyst invasion	32
hsa-miR-125b-5p	Blastocyst	Endometrium	Enhances blastocyst invasion	32
